# The Opium Habit

**Published:** 1876-04

**Authors:** A. A. Lyon

**Affiliations:** Shreeveport, Louisiana


### THE OPIUM HABIT.

By A. A. LYON, M.D., Shreeveport, Louisiana.

While preachers preach, lecturers talk, editors write, and women crusade against the intemperance of whisky drinking, and while all grow justly eloquent in picturing its woes and urging reform, comparately little is seemingly thought and said of that other form of intemperance and dissipation, "opium eating," as it is popularly termed, by which is meant the prostitution of this God-given remedy to the purpose of intoxication. The victims of this pernicious habit may be divided into two classes. First, those hapless invalids, who, with good intent, begin the use of opium legitimately, and under the direction of their physicians, but by degrees have become unconsciously enslaved, and only awake to a realization of their state when too late to retrace. Second, that large class who take it for the enjoyment of that pleasure, psychical and physical, which the peculiar intoxicating properties of the drug afford. This method of intoxication may be preferred because it is more convenient, may be indulged in more secretly, and hence with less liability to discovery than the habit of drinking. But, without stopping to dwell upon the distinctions that may exist between these two classes, but coming straight to the point, we are compelled to admit, however unpleasant the conclusion, that it is an evil fearfully on the increase, and doubtless prevails to an extent that would astound the world, could accurate statistics be obtained and made public.

As between the two evils of opium and alcohol, we regard the latter as the less objectionable, if possible, inasmuch as it is perhaps not only less destructive in its immediate effects, but also because the habit, when fixed, seems less uncontrollable than that of opium, and hence it affords greater hope of reformation. There is to our mind something more repulsively shocking in the ghastly spectre of the opium habit than in the bloated fiery demon of drunkenness; and while it is not to be presumed that the prevalence of opium eating is nearly so extensive in this country as that of drinking, we still have ample proof that it exists to a degree well calculated to excite the gravest apprehensions in the mind of every lover of his race, and painfully to suggest the question: What must be done?

As has been intimated, comparatively little attention has heretofore been given to this subject. It is gratifying, however, to perceive that of late medical men seem to be awakening to the necessities of the situation, and discussion is becoming common among them, and we are not witnout hope that good may come of it, and that some radical measures may be adopted for the cure of this malady that preys not only upon the physical and mental organization, but upon the social and moral also.

It is an every-day thing to hear the profession sweepingly charged with the responsibility of making opium eaters. We emphatically dissent from this view in the great majority of cases, and while no doubt abuses have grown out of the proper use of the drug in some instances, we are wholly unwilling to lay at the feet of the physician what legitimately rests elsewhere. We are satisfied that doctors are often made scapegoats for the convenience for these unfortunate habitues, who themselves, ashamed to assume the responsibility of their deception and disgrace, are always too ready to ascribe to their physician what really springs from their own moral weakness and self-indulgence. Still it is true that we, as practitioners of medicine, are in a position to exert a more powerful influence against the contraction of this habit than any other class of persons. What, then, is our duty in the premises; to banish opium from our practice? Not so. We do not feel that we could practice medicine without it. In our experience of nearly fifteen years, it has ever been a "sine qua non" with us. No remedy in our pharmacopia is perhaps so indispensible. We have never been without it, and hope never to be. We have given it countless numbers of times, and have yet to learn of a single instance where the habit was engendered through our prescriptions. We do not claim that it is not possible. Such result might follow the most careful practice, yet with proper precautions, it need not occur once in a thousand times. But we would scrupulously avoid encouraging any carelessness on this subject, or say a word to abate fear of the danger always in the foreground, but on the contrary, would seek to impress upon the mind of every M.D. who prescribes it, the necessessity of closely attending to and watching the effects of his course. Let him bring to bear upon the mind of his patient the strongest moral influence. Keeping ever before him the horrors of the habit, and the possibility of acquiring it to his utter undoing. In brief, let the physician use his opium as he does fire—never forgetting that, like fire, it is the most serviceable of servants, yet the most tyrannical of tyrants.

Thus the physician may do much to prevent the development of this vice; but to the druggist and apothecary especially must we look to aid in its final arrest, for it is he that holds and dispenses the agent for evil as well as for good. The enlightened spirit of this enlightened country does not recognize opium as an article of common traffic, except in its milder forms and combinations, to be sold without restrictions, at will and for any purpose, as tar, molasses, beeswax, lard, etc., but as a powerful and poisonous drug, presumably kept by the apothecary for medicinal use alone, and *ouly* as medicine should it ever be sold. We are unable to draw the moral distinction between the sale of a drachm of morphine to a notorious opium eater, and a dram of whisky to the staggering sot. If anything, the advantage rests with the bar-keeper, for he professes nothing better, while the druggist seemingly abuses his privilege, and departs from what in his heart he feels to be his duty.

Glaze it over as you may, the fact is still apparent that to-day numberless first-class drug stores make their thousands every year by furnishing, on demand, and without a question, this deadly, damning poison to any and to all ready and willing to pay for it. Thus our druggists become trafficers in the happiness and lives of their fellow-men, and flourish upon the ruin they work. We do not make this statement in a spirit of unqualified condemnation, for we well know the difficulty oftentimes of drawing the line of demarcation between the legitimate and illegitimate sale of the drug, and the perplexity that must arise. But we feel safe in saying, in this connection, that the love of gain too often blinds the eyes and blunts the conscience of many a good man. To all such, and to all physicians, who feel that they have been indiscreet in the use of opium, we would say pause and reflect upon possible, yea probable, results. Can there be any object more pitiable than the doomed opium eater; doomed to a vice that sickens, shrivels and mummifies the body; obliterates every moral faculty, deranges and destroys the intellect, rendering absolutely powerless the will to resist this morbid and insatiable craving? *Everything*—self-respect, honor, religion, friends, home and even tne daily necessities of helpless little children are sacrificed, for the gratification of their appetite—if appetite it may be termed. In drunken stupor the opium eater lies for hours, it may be for days, and with slight intervals, for weeks unconscious of his existence. He awakes miserable, and when occasion brings him into public view, he moves silently among his fellows, with trembling and emaciated form, faltering step, sallow cheeks, expressionless eye, an animated *thing*, aimless and hopeless, seeing nothing, caring for nothing, save this one suicidal indulgence, until at last human nature, unable to endure a tax so prolonged and so terrible, yields, and the wretched victim totters into a premature grave, chiefly remembered among men as the degraded victim of his depraved appetite.

